# 3-(4-Bromo­phenyl­sulfin­yl)-5-fluoro-2-methyl-1-benzofuran

**DOI:** 10.1107/S1600536812005715

**Published:** 2012-02-17

**Authors:** Hong Dae Choi, Pil Ja Seo, Uk Lee

**Affiliations:** a Department of Chemistry, Dongeui University, San 24 Kaya-dong Busanjin-gu, Busan 614-714, Republic of Korea; b Department of Chemistry, Pukyong National University, 599-1 Daeyeon 3-dong, Nam-gu, Busan 608-737, Republic of Korea

## Abstract

There are two symmetry-independent mol­ecules, *A* and *B*, in the asymmetric unit of the title compound, C_15_H_10_BrFO_2_S. The dihedral angle formed by the 4-bromo­phenyl ring and the mean plane of the benzofuran fragment is 88.26 (6)° in mol­ecule *A* and 88.25 (6)° in mol­ecule *B*. In the crystal, mol­ecules are linked by weak inter­molecular C—H⋯F, C—H⋯O and C—H⋯π inter­actions. The crystal structure also exhibits inter­molecular C—Br⋯π [3.737 (3) Å] inter­actions, and weak π–π inter­actions between the benzene and furan rings of neighbouring mol­ecules [centroid–centroid distance = 3.557 (3) Å, inter­planar distance = 3.421 (3) Å and slippage = 0.974 (3) Å].

## Related literature

For the pharmacological activity of benzofuran compounds, see: Aslam *et al.* (2009 ▶); Galal *et al.* (2009 ▶); Khan *et al.* (2005 ▶). For natural products with benzofuran rings, see: Akgul & Anil (2003 ▶); Soekamto *et al.* (2003 ▶). For the crystal structures of related compounds, see: Choi *et al.* (2010*a* ▶,*b* ▶,*c* ▶).
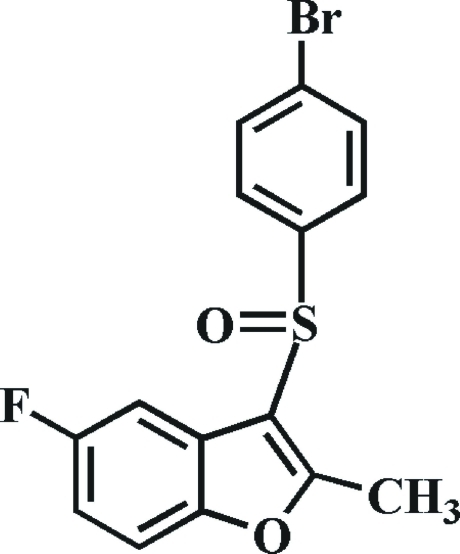


## Experimental

### 

#### Crystal data

C_15_H_10_BrFO_2_S
*M* *_r_* = 353.20
Triclinic, 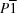

*a* = 9.6576 (1) Å
*b* = 11.9134 (2) Å
*c* = 13.5395 (2) Å
α = 94.038 (1)°
β = 101.096 (1)°
γ = 112.610 (1)°
*V* = 1393.13 (3) Å^3^
*Z* = 4
Mo *K*α radiation
μ = 3.11 mm^−1^
*T* = 173 K
0.34 × 0.27 × 0.17 mm

#### Data collection

Bruker SMART APEXII CCD diffractometer
Absorption correction: multi-scan (*SADABS*; Bruker, 2009 ▶) *T*_min_ = 0.554, *T*_max_ = 0.746
24788 measured reflections
6444 independent reflections
4958 reflections with *I* > 2σ(*I*)
*R*_int_ = 0.039

#### Refinement

*R*[*F* ^2^ > 2σ(*F* ^2^)] = 0.035
*wR*(*F* ^2^) = 0.087
*S* = 1.02
6444 reflections
363 parameters
H-atom parameters constrained
Δρ_max_ = 0.49 e Å^−3^
Δρ_min_ = −0.73 e Å^−3^

### 

Data collection: *APEX2* (Bruker, 2009 ▶); cell refinement: *SAINT* (Bruker, 2009 ▶); data reduction: *SAINT*; program(s) used to solve structure: *SHELXS97* (Sheldrick, 2008 ▶); program(s) used to refine structure: *SHELXL97* (Sheldrick, 2008 ▶); molecular graphics: *ORTEP-3* (Farrugia, 1997 ▶) and *DIAMOND* (Brandenburg, 1998 ▶); software used to prepare material for publication: *SHELXL97*.

## Supplementary Material

Crystal structure: contains datablock(s) global, I. DOI: 10.1107/S1600536812005715/zs2180sup1.cif

Structure factors: contains datablock(s) I. DOI: 10.1107/S1600536812005715/zs2180Isup2.hkl

Supplementary material file. DOI: 10.1107/S1600536812005715/zs2180Isup3.cml

Additional supplementary materials:  crystallographic information; 3D view; checkCIF report

## Figures and Tables

**Table 1:** Hydrogen-bond geometry (Å, °) *Cg*1 is the centroid of the C17–C22 benzene ring.

*D*—H⋯*A*	*D*—H	H⋯*A*	*D*⋯*A*	*D*—H⋯*A*
C3—H3⋯F1^i^	0.95	2.44	3.316 (3)	154
C15—H15⋯O4^ii^	0.95	2.42	3.323 (3)	158
C24—H24*C*⋯O2^iii^	0.98	2.59	3.502 (3)	154
C5—H5⋯*Cg*1^iv^	0.95	2.74	3.612 (3)	154

Symmetry codes: (i) 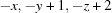; (ii) 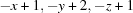; (iii) 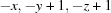; (iv) 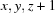.

**Fig. 1.**
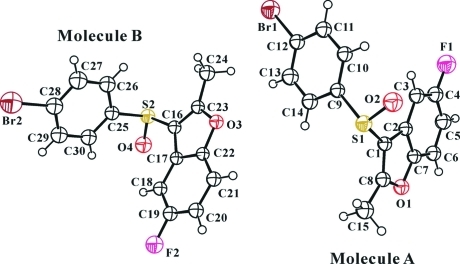
The molecular structure of the title compound with the atom numbering scheme. Displacement ellipsoids are drawn at the 50% probability level. H atoms are presented as small spheres of arbitrary radius.

**Fig. 2.**
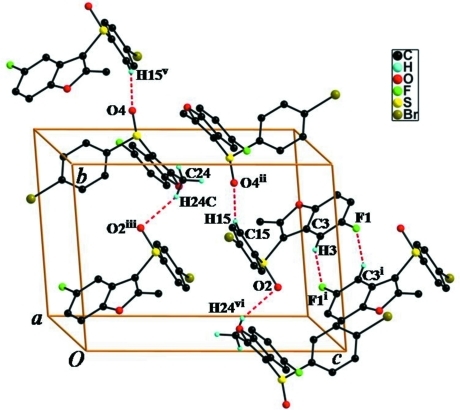
A view of the C–H···O and C–H···F interactions (dotted lines) in the crystal structure of the title compound. H atoms non-participating in hydrogen-bonding were omitted for clarity. For symmetry codes: (v) - x + 1, - y + 2, - z + 1; (vi) - x, - y + 1, - z + 1]. For other codes, see Table 1.

**Fig. 3.**
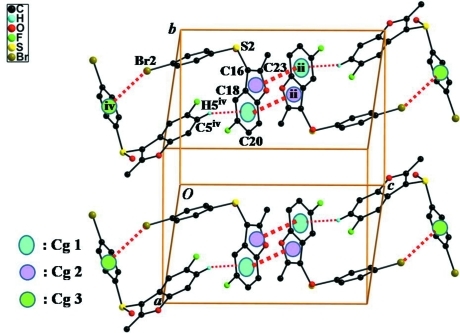
A view of the C–H···π, C–Br···π and π–π interactions (dotted lines) in the crystal structure of the title compound. H atoms non-participating in hydrogen-bonding were omitted for clarity. For symmetry codes, see Table 1.

## supplementary crystallographic information

### Comment

Benzofuran derivatives have drawn considerable attention owing to their valuable biological properties such as antibacterial and antifungal, antitumor and antiviral, and antimicrobial activities (Aslam *et al.*, 2009, Galal *et al.*, 2009, Khan *et al.*, 2005). These benzofuran derivatives occur in a wide range of natural products (Akgul & Anil, 2003; Soekamto *et al.*, 2003). As a part of our continuing study of 5-fluoro-2-methyl-1-benzofuran analoques containing 3-phenylsulfonyl (Choi *et al.*, 2010*a*), 3-(4-fluorophenylsulfinyl) (Choi *et al.*, 2010*b*) and 3-(4-fluorophenylsufonyl) (Choi *et al.*, 2010*c*) substituents, we report herein the crystal structure of the title compound which crystallizes with two unique molecule, A & B, in the asymmetric unit.

In the title molecule (Fig. 1), the benzofuran unit is essentially planar, with a mean deviation of 0.002 (2) Å, for A, and 0.009 (2) Å, for B, respectively, from the least-squares plane defined by the nine constituent atoms. The dihedral angles between the 4-bromophenyl ring and the mean plane of the benzofurn fragment are 88.26 (6)° in molecule A and 88.25 (6)° in molecule B, respectively. The crystal packing (Fig. 2) is stabilized by weak intermolecular C–H···F (Table 1; first entry) and C–H···O (Table 1; second and third entry) hydrogen bonds. The crystal packing (Fig. 3) is further stabilized by intermolecular C–H···π interactions (Table 1; fourth entry, Cg1 is the centroid of the C17-C22 benzene ring), and by intermolecular C28–Br2···π interactions between the bromine atom and the 4-bromophenyl ring of a neighbouring molecule with Br2···Cg3^iv^ being 3.737 (3) Å (Cg3 is the centroid of the C9–C14 4-bromophenyl ring). Additionally, the crystal packing (Fig. 3) exhibits weak slipped π–π interactions between the benzene and furan rings of neighbouring molecules, with a Cg1···Cg2^ii^ distance of 3.557 (3) Å and an interplanar distance of 3.421 (3) Å resulting in a slippage of 0.974 (3) Å (Cg2 is the centroid of the C1/C2/C7/O1/C8 furan ring).

### Experimental

3-Chloroperoxybenzoic acid (77%) (224 mg, 1.0 mmol) was added in small portions to a stirred solution of 3-(4-bromophenylsulfanyl)-5-fluoro-2-methyl-1-benzofuran (303 mg, 0.9 mmol) in dichloromethane (40 mL) at 273 K. After being stirred at room temperature for 5h, the mixture was washed with saturated sodium bicarbonate solution and the organic layer was separated, dried over magnesium sulfate, filtered and concentrated at reduced pressure. The residue was purified by column chromatography (hexane-ethyl acetate, 2:1 v/v) to afford the title compound as a colorless solid [yield 71%, m.p. 382-383 K; *R*_f_ = 0.45 (hexane-ethyl acetate, 2:1 v/v)]. Single crystals suitable for X-ray diffraction were prepared by slow evaporation of a solution of the title compound in acetone at room temperature.

### Refinement

All H atoms were positioned geometrically and refined using a riding model, with C–H = 0.95 Å for aryl and 0.98 Å for methyl H atoms. *U*_iso_(H) = 1.2*U*_eq_(C) for aryl and 1.5*U*_eq_(C) for methyl H atoms.

### Crystal data

**Table:**

C_15_H_10_BrFO_2_S	*Z* = 4
*M**_r_* = 353.20	*F*(000) = 704
Triclinic, *P*1	*D*_x_ = 1.684 Mg m^−^^3^
Hall symbol: -P 1	Melting point = 382–383 K
*a* = 9.6576 (1) Å	Mo *K*α radiation, λ = 0.71073 Å
*b* = 11.9134 (2) Å	Cell parameters from 8290 reflections
*c* = 13.5395 (2) Å	θ = 2.2–27.2°
α = 94.038 (1)°	µ = 3.11 mm^−^^1^
β = 101.096 (1)°	*T* = 173 K
γ = 112.610 (1)°	Block, colourless
*V* = 1393.13 (3) Å^3^	0.34 × 0.27 × 0.17 mm

### Data collection

**Table:**

Bruker SMART APEXII CCD diffractometer	6444 independent reflections
Radiation source: rotating anode	4958 reflections with *I* > 2σ(*I*)
Graphite multilayer monochromator	*R*_int_ = 0.039
Detector resolution: 10.0 pixels mm^-1^	θ_max_ = 27.6°, θ_min_ = 1.6°
φ and ω scans	*h* = −12→12
Absorption correction: multi-scan (*SADABS*; Bruker, 2009)	*k* = −15→15
*T*_min_ = 0.554, *T*_max_ = 0.746	*l* = −17→17
24788 measured reflections	

### Refinement

**Table:**

Refinement on *F*^2^	Primary atom site location: structure-invariant direct methods
Least-squares matrix: full	Secondary atom site location: difference Fourier map
*R*[*F*^2^ > 2σ(*F*^2^)] = 0.035	Hydrogen site location: difference Fourier map
*wR*(*F*^2^) = 0.087	H-atom parameters constrained
*S* = 1.02	*w* = 1/[σ^2^(*F*_o_^2^) + (0.0348*P*)^2^ + 0.8229*P*] where *P* = (*F*_o_^2^ + 2*F*_c_^2^)/3
6444 reflections	(Δ/σ)_max_ = 0.001
363 parameters	Δρ_max_ = 0.49 e Å^−^^3^
0 restraints	Δρ_min_ = −0.73 e Å^−^^3^

### Special details

**Table:**

Geometry. All esds (except the esd in the dihedral angle between two l.s. planes) are estimated using the full covariance matrix. The cell esds are taken into account individually in the estimation of esds in distances, angles and torsion angles; correlations between esds in cell parameters are only used when they are defined by crystal symmetry. An approximate (isotropic) treatment of cell esds is used for estimating esds involving l.s. planes.
Refinement. Refinement of F^2^ against ALL reflections. The weighted R-factor wR and goodness of fit S are based on F^2^, conventional R-factors R are based on F, with F set to zero for negative F^2^. The threshold expression of F^2^ > 2sigma(F^2^) is used only for calculating R-factors(gt) etc. and is not relevant to the choice of reflections for refinement. R-factors based on F^2^ are statistically about twice as large as those based on F, and R- factors based on ALL data will be even larger.

### Fractional atomic coordinates and isotropic or equivalent isotropic displacement parameters (Å^2^)

**Table:**

	*x*	*y*	*z*	*U*_iso_*/*U*_eq_	
Br1	−0.19888 (4)	0.64684 (3)	0.55467 (2)	0.05801 (11)	
S1	0.27869 (7)	0.41871 (5)	0.74250 (5)	0.03725 (14)	
O1	0.62608 (18)	0.66639 (14)	0.94699 (13)	0.0358 (4)	
O2	0.1845 (2)	0.30693 (15)	0.78067 (15)	0.0497 (5)	
F1	0.12944 (19)	0.63331 (15)	1.09105 (13)	0.0607 (5)	
C1	0.4009 (3)	0.5329 (2)	0.84628 (17)	0.0305 (5)	
C2	0.3666 (3)	0.58567 (19)	0.93269 (16)	0.0282 (4)	
C3	0.2340 (3)	0.5723 (2)	0.96518 (18)	0.0359 (5)	
H3	0.1341	0.5171	0.9271	0.043*	
C4	0.2560 (3)	0.6437 (2)	1.05536 (19)	0.0392 (5)	
C5	0.3978 (3)	0.7245 (2)	1.11426 (18)	0.0406 (6)	
H5	0.4044	0.7711	1.1762	0.049*	
C6	0.5302 (3)	0.7375 (2)	1.08299 (18)	0.0376 (5)	
H6	0.6297	0.7920	1.1222	0.045*	
C7	0.5101 (3)	0.66719 (19)	0.99181 (17)	0.0309 (5)	
C8	0.5566 (3)	0.5840 (2)	0.85873 (18)	0.0335 (5)	
C9	0.6600 (3)	0.5691 (3)	0.7962 (2)	0.0472 (6)	
H15A	0.7009	0.6429	0.7647	0.071*	
H15B	0.6019	0.4970	0.7428	0.071*	
H15C	0.7457	0.5577	0.8396	0.071*	
C10	0.1497 (3)	0.4894 (2)	0.69659 (16)	0.0312 (5)	
C11	−0.0031 (3)	0.4298 (2)	0.69762 (18)	0.0351 (5)	
H11	−0.0371	0.3567	0.7269	0.042*	
C12	−0.1076 (3)	0.4776 (2)	0.65540 (18)	0.0372 (5)	
H12	−0.2135	0.4382	0.6562	0.045*	
C13	−0.0552 (3)	0.5827 (2)	0.61246 (17)	0.0366 (5)	
C14	0.0986 (3)	0.6429 (2)	0.61141 (18)	0.0417 (6)	
H14	0.1325	0.7160	0.5821	0.050*	
C15	0.2025 (3)	0.5955 (2)	0.65345 (18)	0.0389 (5)	
H15	0.3084	0.6350	0.6528	0.047*	
Br2	0.08498 (5)	0.79746 (3)	−0.15733 (2)	0.07262 (12)	
S2	0.28070 (7)	1.09998 (5)	0.29462 (5)	0.03822 (15)	
O3	0.28658 (18)	0.82877 (14)	0.44327 (12)	0.0327 (3)	
O4	0.4310 (2)	1.20632 (15)	0.30530 (15)	0.0517 (5)	
F2	0.80169 (17)	0.96487 (15)	0.29759 (12)	0.0505 (4)	
C16	0.3202 (3)	0.98537 (19)	0.35506 (17)	0.0297 (5)	
C17	0.4479 (2)	0.95086 (18)	0.35492 (16)	0.0275 (4)	
C18	0.5797 (3)	0.9915 (2)	0.31620 (17)	0.0316 (5)	
H18	0.6039	1.0589	0.2796	0.038*	
C19	0.6722 (3)	0.9282 (2)	0.33421 (18)	0.0342 (5)	
C20	0.6434 (3)	0.8299 (2)	0.38744 (18)	0.0357 (5)	
H20	0.7118	0.7896	0.3965	0.043*	
C21	0.5147 (3)	0.7908 (2)	0.42724 (17)	0.0345 (5)	
H21	0.4921	0.7240	0.4647	0.041*	
C22	0.4202 (2)	0.85308 (19)	0.41017 (16)	0.0287 (5)	
C23	0.2295 (2)	0.91161 (19)	0.40823 (17)	0.0307 (5)	
C24	0.0814 (3)	0.9007 (2)	0.4318 (2)	0.0407 (6)	
H24A	0.0626	0.9736	0.4169	0.061*	
H24B	0.0869	0.8945	0.5041	0.061*	
H24C	−0.0031	0.8269	0.3900	0.061*	
C25	0.2258 (3)	1.0190 (2)	0.16674 (19)	0.0357 (5)	
C26	0.0802 (3)	0.9237 (2)	0.1333 (2)	0.0427 (6)	
H26	0.0107	0.9038	0.1767	0.051*	
C27	0.0376 (3)	0.8582 (2)	0.0363 (2)	0.0495 (7)	
H27	−0.0615	0.7925	0.0122	0.059*	
C28	0.1403 (3)	0.8894 (2)	−0.0250 (2)	0.0459 (6)	
C29	0.2847 (3)	0.9855 (2)	0.0071 (2)	0.0462 (6)	
H29	0.3537	1.0057	−0.0367	0.055*	
C30	0.3269 (3)	1.0515 (2)	0.1042 (2)	0.0414 (6)	
H30	0.4248	1.1188	0.1274	0.050*	

### Atomic displacement parameters (Å^2^)

**Table:**

	*U*^11^	*U*^22^	*U*^33^	*U*^12^	*U*^13^	*U*^23^
Br1	0.0696 (2)	0.0880 (2)	0.04255 (16)	0.05785 (19)	0.01397 (14)	0.01981 (14)
S1	0.0372 (3)	0.0393 (3)	0.0347 (3)	0.0205 (3)	0.0007 (3)	−0.0048 (2)
O1	0.0243 (8)	0.0350 (8)	0.0434 (9)	0.0086 (7)	0.0046 (7)	0.0069 (7)
O2	0.0450 (11)	0.0296 (8)	0.0623 (12)	0.0122 (8)	−0.0068 (9)	0.0031 (8)
F1	0.0488 (10)	0.0662 (10)	0.0585 (10)	0.0102 (8)	0.0284 (8)	−0.0102 (8)
C1	0.0279 (11)	0.0346 (11)	0.0290 (11)	0.0135 (9)	0.0048 (9)	0.0058 (9)
C2	0.0289 (11)	0.0261 (10)	0.0261 (10)	0.0089 (9)	0.0032 (9)	0.0052 (8)
C3	0.0259 (12)	0.0381 (12)	0.0338 (12)	0.0045 (10)	0.0050 (10)	0.0000 (10)
C4	0.0362 (13)	0.0401 (13)	0.0379 (13)	0.0092 (11)	0.0154 (11)	0.0035 (10)
C5	0.0509 (16)	0.0361 (12)	0.0276 (12)	0.0124 (12)	0.0065 (11)	−0.0001 (10)
C6	0.0374 (13)	0.0305 (11)	0.0336 (12)	0.0075 (10)	−0.0022 (10)	0.0020 (9)
C7	0.0272 (11)	0.0284 (10)	0.0342 (12)	0.0100 (9)	0.0025 (9)	0.0089 (9)
C8	0.0331 (12)	0.0332 (11)	0.0389 (12)	0.0171 (10)	0.0088 (10)	0.0131 (10)
C9	0.0398 (15)	0.0505 (15)	0.0619 (18)	0.0232 (13)	0.0228 (13)	0.0177 (13)
C10	0.0335 (12)	0.0347 (11)	0.0228 (10)	0.0147 (10)	0.0013 (9)	−0.0024 (9)
C11	0.0367 (13)	0.0334 (11)	0.0342 (12)	0.0146 (10)	0.0072 (10)	0.0012 (9)
C12	0.0296 (12)	0.0421 (13)	0.0375 (13)	0.0143 (11)	0.0060 (10)	−0.0016 (10)
C13	0.0452 (14)	0.0496 (14)	0.0235 (11)	0.0304 (12)	0.0043 (10)	0.0036 (10)
C14	0.0516 (16)	0.0463 (14)	0.0338 (13)	0.0231 (13)	0.0155 (12)	0.0137 (11)
C15	0.0362 (13)	0.0467 (14)	0.0347 (12)	0.0167 (11)	0.0105 (10)	0.0073 (11)
Br2	0.0903 (3)	0.0663 (2)	0.03856 (16)	0.01303 (19)	0.00546 (16)	0.00434 (14)
S2	0.0365 (3)	0.0280 (3)	0.0498 (4)	0.0175 (2)	0.0019 (3)	0.0015 (2)
O3	0.0291 (8)	0.0357 (8)	0.0324 (8)	0.0132 (7)	0.0063 (7)	0.0034 (6)
O4	0.0482 (11)	0.0252 (8)	0.0664 (12)	0.0073 (8)	−0.0022 (9)	0.0010 (8)
F2	0.0352 (8)	0.0640 (10)	0.0606 (10)	0.0256 (7)	0.0184 (7)	0.0110 (8)
C16	0.0281 (11)	0.0249 (10)	0.0314 (11)	0.0116 (9)	−0.0018 (9)	−0.0043 (8)
C17	0.0261 (11)	0.0257 (10)	0.0255 (10)	0.0100 (9)	−0.0010 (8)	−0.0054 (8)
C18	0.0276 (12)	0.0311 (11)	0.0331 (12)	0.0113 (9)	0.0034 (9)	0.0008 (9)
C19	0.0246 (11)	0.0410 (12)	0.0340 (12)	0.0131 (10)	0.0039 (9)	−0.0033 (10)
C20	0.0329 (13)	0.0405 (12)	0.0356 (12)	0.0237 (11)	−0.0022 (10)	−0.0045 (10)
C21	0.0370 (13)	0.0344 (11)	0.0316 (12)	0.0179 (10)	0.0004 (10)	0.0030 (9)
C22	0.0265 (11)	0.0301 (10)	0.0255 (10)	0.0109 (9)	0.0009 (9)	−0.0030 (8)
C23	0.0260 (11)	0.0301 (10)	0.0299 (11)	0.0103 (9)	−0.0010 (9)	−0.0066 (9)
C24	0.0279 (12)	0.0470 (14)	0.0439 (14)	0.0140 (11)	0.0078 (11)	−0.0040 (11)
C25	0.0324 (12)	0.0298 (11)	0.0430 (13)	0.0142 (10)	−0.0002 (10)	0.0101 (10)
C26	0.0319 (13)	0.0458 (14)	0.0424 (14)	0.0091 (11)	0.0044 (11)	0.0097 (11)
C27	0.0375 (15)	0.0456 (14)	0.0453 (15)	0.0013 (12)	−0.0023 (12)	0.0077 (12)
C28	0.0537 (17)	0.0392 (13)	0.0371 (13)	0.0148 (12)	0.0006 (12)	0.0097 (11)
C29	0.0460 (16)	0.0464 (14)	0.0451 (15)	0.0154 (13)	0.0120 (12)	0.0181 (12)
C30	0.0328 (13)	0.0327 (12)	0.0518 (15)	0.0073 (10)	0.0052 (12)	0.0129 (11)

### Geometric parameters (Å, º)

**Table:**

Br1—C13	1.898 (2)	Br2—C28	1.897 (3)
S1—O2	1.4881 (19)	S2—O4	1.4876 (19)
S1—C1	1.754 (2)	S2—C16	1.766 (2)
S1—C10	1.797 (2)	S2—C25	1.797 (3)
O1—C8	1.364 (3)	O3—C23	1.374 (3)
O1—C7	1.375 (3)	O3—C22	1.380 (3)
F1—C4	1.365 (3)	F2—C19	1.360 (3)
C1—C8	1.358 (3)	C16—C23	1.341 (3)
C1—C2	1.442 (3)	C16—C17	1.442 (3)
C2—C3	1.388 (3)	C17—C18	1.395 (3)
C2—C7	1.391 (3)	C17—C22	1.396 (3)
C3—C4	1.369 (3)	C18—C19	1.373 (3)
C3—H3	0.9500	C18—H18	0.9500
C4—C5	1.377 (4)	C19—C20	1.383 (3)
C5—C6	1.379 (4)	C20—C21	1.379 (3)
C5—H5	0.9500	C20—H20	0.9500
C6—C7	1.379 (3)	C21—C22	1.379 (3)
C6—H6	0.9500	C21—H21	0.9500
C8—C9	1.479 (3)	C23—C24	1.484 (3)
C9—H15A	0.9800	C24—H24A	0.9800
C9—H15B	0.9800	C24—H24B	0.9800
C9—H15C	0.9800	C24—H24C	0.9800
C10—C11	1.373 (3)	C25—C30	1.377 (4)
C10—C15	1.386 (3)	C25—C26	1.388 (3)
C11—C12	1.391 (3)	C26—C27	1.380 (4)
C11—H11	0.9500	C26—H26	0.9500
C12—C13	1.375 (3)	C27—C28	1.374 (4)
C12—H12	0.9500	C27—H27	0.9500
C13—C14	1.382 (4)	C28—C29	1.384 (4)
C14—C15	1.382 (4)	C29—C30	1.383 (4)
C14—H14	0.9500	C29—H29	0.9500
C15—H15	0.9500	C30—H30	0.9500
			
O2—S1—C1	109.43 (11)	O4—S2—C16	107.26 (11)
O2—S1—C10	105.77 (11)	O4—S2—C25	107.02 (12)
C1—S1—C10	98.65 (10)	C16—S2—C25	96.12 (10)
C8—O1—C7	106.70 (17)	C23—O3—C22	106.26 (17)
C8—C1—C2	107.5 (2)	C23—C16—C17	107.87 (19)
C8—C1—S1	121.82 (18)	C23—C16—S2	124.12 (17)
C2—C1—S1	130.60 (17)	C17—C16—S2	127.99 (18)
C3—C2—C7	119.7 (2)	C18—C17—C22	119.4 (2)
C3—C2—C1	135.9 (2)	C18—C17—C16	136.2 (2)
C7—C2—C1	104.4 (2)	C22—C17—C16	104.42 (19)
C4—C3—C2	115.9 (2)	C19—C18—C17	116.0 (2)
C4—C3—H3	122.0	C19—C18—H18	122.0
C2—C3—H3	122.0	C17—C18—H18	122.0
C3—C4—F1	118.2 (2)	F2—C19—C18	117.8 (2)
C3—C4—C5	124.7 (2)	F2—C19—C20	117.6 (2)
F1—C4—C5	117.1 (2)	C18—C19—C20	124.6 (2)
C4—C5—C6	119.8 (2)	C21—C20—C19	119.5 (2)
C4—C5—H5	120.1	C21—C20—H20	120.2
C6—C5—H5	120.1	C19—C20—H20	120.2
C5—C6—C7	116.4 (2)	C20—C21—C22	116.9 (2)
C5—C6—H6	121.8	C20—C21—H21	121.6
C7—C6—H6	121.8	C22—C21—H21	121.6
O1—C7—C6	125.7 (2)	C21—C22—O3	126.0 (2)
O1—C7—C2	110.70 (19)	C21—C22—C17	123.6 (2)
C6—C7—C2	123.6 (2)	O3—C22—C17	110.39 (18)
C1—C8—O1	110.7 (2)	C16—C23—O3	111.06 (19)
C1—C8—C9	133.1 (2)	C16—C23—C24	132.7 (2)
O1—C8—C9	116.3 (2)	O3—C23—C24	116.2 (2)
C8—C9—H15A	109.5	C23—C24—H24A	109.5
C8—C9—H15B	109.5	C23—C24—H24B	109.5
H15A—C9—H15B	109.5	H24A—C24—H24B	109.5
C8—C9—H15C	109.5	C23—C24—H24C	109.5
H15A—C9—H15C	109.5	H24A—C24—H24C	109.5
H15B—C9—H15C	109.5	H24B—C24—H24C	109.5
C11—C10—C15	121.6 (2)	C30—C25—C26	121.3 (2)
C11—C10—S1	118.17 (18)	C30—C25—S2	120.24 (18)
C15—C10—S1	119.95 (18)	C26—C25—S2	118.5 (2)
C10—C11—C12	119.4 (2)	C27—C26—C25	119.3 (3)
C10—C11—H11	120.3	C27—C26—H26	120.3
C12—C11—H11	120.3	C25—C26—H26	120.3
C13—C12—C11	118.9 (2)	C28—C27—C26	119.1 (2)
C13—C12—H12	120.5	C28—C27—H27	120.5
C11—C12—H12	120.5	C26—C27—H27	120.5
C14—C13—C12	121.8 (2)	C27—C28—C29	122.0 (3)
C14—C13—Br1	119.60 (18)	C27—C28—Br2	119.5 (2)
C12—C13—Br1	118.60 (19)	C29—C28—Br2	118.5 (2)
C13—C14—C15	119.3 (2)	C30—C29—C28	118.8 (3)
C13—C14—H14	120.4	C30—C29—H29	120.6
C15—C14—H14	120.4	C28—C29—H29	120.6
C14—C15—C10	119.0 (2)	C25—C30—C29	119.4 (2)
C14—C15—H15	120.5	C25—C30—H30	120.3
C10—C15—H15	120.5	C29—C30—H30	120.3
			
O2—S1—C1—C8	122.96 (19)	O4—S2—C16—C23	143.29 (19)
C10—S1—C1—C8	−126.85 (19)	C25—S2—C16—C23	−106.7 (2)
O2—S1—C1—C2	−53.8 (2)	O4—S2—C16—C17	−38.7 (2)
C10—S1—C1—C2	56.4 (2)	C25—S2—C16—C17	71.3 (2)
C8—C1—C2—C3	−179.5 (3)	C23—C16—C17—C18	−178.5 (2)
S1—C1—C2—C3	−2.5 (4)	S2—C16—C17—C18	3.2 (4)
C8—C1—C2—C7	0.1 (2)	C23—C16—C17—C22	0.7 (2)
S1—C1—C2—C7	177.15 (17)	S2—C16—C17—C22	−177.64 (16)
C7—C2—C3—C4	0.5 (3)	C22—C17—C18—C19	1.5 (3)
C1—C2—C3—C4	−179.9 (2)	C16—C17—C18—C19	−179.4 (2)
C2—C3—C4—F1	−179.5 (2)	C17—C18—C19—F2	179.93 (19)
C2—C3—C4—C5	−0.6 (4)	C17—C18—C19—C20	−0.4 (3)
C3—C4—C5—C6	0.1 (4)	F2—C19—C20—C21	179.0 (2)
F1—C4—C5—C6	179.0 (2)	C18—C19—C20—C21	−0.7 (4)
C4—C5—C6—C7	0.5 (3)	C19—C20—C21—C22	0.6 (3)
C8—O1—C7—C6	179.6 (2)	C20—C21—C22—O3	−179.97 (19)
C8—O1—C7—C2	0.0 (2)	C20—C21—C22—C17	0.6 (3)
C5—C6—C7—O1	179.9 (2)	C23—O3—C22—C21	−179.3 (2)
C5—C6—C7—C2	−0.6 (3)	C23—O3—C22—C17	0.2 (2)
C3—C2—C7—O1	179.63 (19)	C18—C17—C22—C21	−1.6 (3)
C1—C2—C7—O1	−0.1 (2)	C16—C17—C22—C21	179.0 (2)
C3—C2—C7—C6	0.0 (3)	C18—C17—C22—O3	178.83 (18)
C1—C2—C7—C6	−179.7 (2)	C16—C17—C22—O3	−0.5 (2)
C2—C1—C8—O1	−0.1 (2)	C17—C16—C23—O3	−0.6 (2)
S1—C1—C8—O1	−177.45 (15)	S2—C16—C23—O3	177.83 (14)
C2—C1—C8—C9	−178.9 (2)	C17—C16—C23—C24	−178.0 (2)
S1—C1—C8—C9	3.7 (4)	S2—C16—C23—C24	0.4 (4)
C7—O1—C8—C1	0.0 (2)	C22—O3—C23—C16	0.2 (2)
C7—O1—C8—C9	179.08 (19)	C22—O3—C23—C24	178.11 (18)
O2—S1—C10—C11	−4.0 (2)	O4—S2—C25—C30	7.5 (2)
C1—S1—C10—C11	−117.14 (19)	C16—S2—C25—C30	−102.7 (2)
O2—S1—C10—C15	−178.55 (18)	O4—S2—C25—C26	−172.55 (18)
C1—S1—C10—C15	68.3 (2)	C16—S2—C25—C26	77.3 (2)
C15—C10—C11—C12	−0.7 (3)	C30—C25—C26—C27	1.7 (4)
S1—C10—C11—C12	−175.12 (17)	S2—C25—C26—C27	−178.2 (2)
C10—C11—C12—C13	0.8 (3)	C25—C26—C27—C28	−0.3 (4)
C11—C12—C13—C14	−0.9 (4)	C26—C27—C28—C29	−0.8 (4)
C11—C12—C13—Br1	179.82 (17)	C26—C27—C28—Br2	178.5 (2)
C12—C13—C14—C15	0.8 (4)	C27—C28—C29—C30	0.3 (4)
Br1—C13—C14—C15	−179.88 (18)	Br2—C28—C29—C30	−178.88 (19)
C13—C14—C15—C10	−0.7 (4)	C26—C25—C30—C29	−2.1 (4)
C11—C10—C15—C14	0.6 (3)	S2—C25—C30—C29	177.82 (19)
S1—C10—C15—C14	174.96 (18)	C28—C29—C30—C25	1.1 (4)

### Hydrogen-bond geometry (Å, º)

Cg1 is the centroid of the C17–C22 benzene ring.

**Table:**

*D*—H···*A*	*D*—H	H···*A*	*D*···*A*	*D*—H···*A*
C3—H3···F1^i^	0.95	2.44	3.316 (3)	154
C15—H15···O4^ii^	0.95	2.42	3.323 (3)	158
C24—H24*C*···O2^iii^	0.98	2.59	3.502 (3)	154
C5—H5···*Cg*1^iv^	0.95	2.74	3.612 (3)	154

Symmetry codes: (i) −*x*, −*y*+1, −*z*+2; (ii) −*x*+1, −*y*+2, −*z*+1; (iii) −*x*, −*y*+1, −*z*+1; (iv) *x*, *y*, *z*+1.
